# Energy Efficiency Maximization for WSNs with Simultaneous Wireless Information and Power Transfer

**DOI:** 10.3390/s17081906

**Published:** 2017-08-18

**Authors:** Hongyan Yu, Yongqiang Zhang, Songtao Guo, Yuanyuan Yang, Luyue Ji

**Affiliations:** 1 College of Electronic and Information Engineering, Southwest University, Chongqing 400715, China; yhy123@email.swu.edu.cn (H.Y.); yqcheung@email.swu.edu.cn (Y.Z.); july117@email.swu.edu.cn (L.J.); 2 Department of Electrical & Computer Engineering, Stony Brook University, Stony Brook, NY 11794, USA

**Keywords:** SWIPT, energy efficiency, energy-spectral efficiency tradeoff, effective throughput, wireless rechargeable sensor networks

## Abstract

Recently, the simultaneous wireless information and power transfer (SWIPT) technique has been regarded as a promising approach to enhance performance of wireless sensor networks with limited energy supply. However, from a green communication perspective, energy efficiency optimization for SWIPT system design has not been investigated in Wireless Rechargeable Sensor Networks (WRSNs). In this paper, we consider the tradeoffs between energy efficiency and three factors including spectral efficiency, the transmit power and outage target rate for two different modes, i.e., power splitting (PS) and time switching modes (TS), at the receiver. Moreover, we formulate the energy efficiency maximization problem subject to the constraints of minimum Quality of Service (QoS), minimum harvested energy and maximum transmission power as non-convex optimization problem. In particular, we focus on optimizing power control and power allocation policy in PS and TS modes to maximize energy efficiency of data transmission. For PS and TS modes, we propose the corresponding algorithm to characterize a non-convex optimization problem that takes into account the circuit power consumption and the harvested energy. By exploiting nonlinear fractional programming and Lagrangian dual decomposition, we propose suboptimal iterative algorithms to obtain the solutions of non-convex optimization problems. Furthermore, we derive the outage probability and effective throughput from the scenarios that the transmitter does not or partially know the channel state information (CSI) of the receiver. Simulation results illustrate that the proposed optimal iterative algorithm can achieve optimal solutions within a small number of iterations and various tradeoffs between energy efficiency and spectral efficiency, transmit power and outage target rate, respectively.

## 1. Introduction

Currently, energy efficiency is an important objective in the analysis and design of wireless sensor networks, in addition to the traditional interest in higher rates and quality of service [1,2,3,4]. According to the technical report from Ericiss [5], by 2020, there will be more than 50 billion connected devices, including sensors, smart phones, medical and wearable devices connected to the Internet. Clearly, in order to serve such a massive number of terminals, future networks will have to dramatically increase the energy consumption compared to the present network. More importantly, most sensors are powered by finite battery capacity. In order to address these issues, wireless power transfer (WPT) is a promising approach to harvest radio frequency (RF)-energy from wireless received signal and prolong the lifework time of wireless sensor networks. Accordingly, the WPT as the wireless charging technology enables an intentional RF power source to transmit electromagnetic energy to an electrical load across the air media without an interconnected line. Due to its convenience and better user experience, some researchers have begun to investigate wireless power transfer algorithms, technologies and applications in wireless sensor networks [6,7,8,9,10,11,12,13,14,15,16,17,18,19,20]. In [6], the network architecture for wireless rechargeable sensor networks (WRSNs) was introduced by describing the functionality of network components and their features. The perpetual operation condition for WRSNs was analyzed and derived. In [7], the abstract model, algorithm design and networking principles about wireless power transfer in ad hoc communication network were introduced. Especially, several applications for wirelessly powered communication networks (WPCN) were presented and the relevant performance tradeoffs were characterized. In [8,9,10,11,12], some works on joint mobile data gathering and energy provisioning in Wireless Rechargeable Sensor Networks (WRSN) were investigated to provide perpetual network operations by capturing renewable energy from external environments. Wireless power transfer provides a promising means of replenishing battery-powered devices and supports various applications [13,14,15,16,17,18,19,20].

On the other hand, since radio signals carry both information and RF energy at the same time, simultaneous wireless information and power transfer (SWIPT) has recently been proposed and attracted much attention from academia and industry [21,22,23,24]. However, it is not realizable that the receiver for SWIPT technology is required to be able to decode information and harvest energy from the same signal, which is due to the practical circuit designment limitations [21]. Therefore, two receiver design schemes, namely power splitting (PS) and time switching (TS), were proposed in [21,23]. In addition, in [21], optimal mode switching rule at the receiver to achieve the tradeoff between wireless information and energy harvesting was derived. In [22], a unified study on SWIPT for a Multiple Input Multiple Output (MIMO) broadcast system was pursued to characterize their achievable rate-energy (R-E) regions for two practical designs, i.e., PS and TS mode. In addition, in [23], a general receiver operation, namely, dymamic power splitting (DPS) was proposed to characterize R-E regions and derive different R-E tradeoffs. In [24], based on the instantaneous channel condition and the proposed DPS scheme, an optimal power splitting rule at the receiver was derived to achieve the tradeoffs between the maximum ergodic capacity and the maximum average harvested energy. However, these works focus on how to achieve rate-energy tradeoff and increase R-E regions and/or maximize transmission rate subject to the harvested energy constraint. The tradeoff between energy efficiency (EE) and spectral efficiency (SE) and the EE optimization problem for SWIPT system have not been considered in detail. In particular, the increasingly strict environmental standard and rapidly rising energy cost have led to an emerging trend of addressing the energy efficiency of wireless networks. Moreover, EE optimization of transceiver design in the SWIPT system has also not been investigated in wireless sensor networks from a green communication perspective.

Recently, the EE optimization problem for the SWIPT system has been considered in [19,25,26,27,28,29,30,31,32,33]. In [19], an energy-efficient cooperative transmission problem for SWIPT in clustered wireless sensor networks was formulated to develop a distributed iteration algorithm for power allocation, power splitting and relay selection. In [25], a resource allocation algorithm for maximization of the energy efficiency of data transmission was investigated in orthogonal frequency division multiple access (OFDMA) systems with SWIPT. Furthermore, in [26], a power splitting based multiuser multiple-input-single-output (MISO) downlink system with SWIPT was considered to maximize the system energy efficiency by joint beamforming and PS schemes. Similarly, in [27], the main objective is to maximize the ratio of the achievable utility to the total power consumption subject to harvested energy requirements and power budget at the base station (BS). Moreover, in [28], two user multiple access channels with SWIPT were studied to optimize resource allocation strategy for SWIPT focusing on the system energy efficiency. In [29], an energy efficient resource allocation scheme for SWIPT with imperfect channel estimation was studied by determining the training interval. Furthermore, in [30], an algorithm for EE optimization was proposed in the MISO system with SWIPT to reveal the influence of the searching interval, channel fading, maximum transmit power and the Quality of Service (QoS) requirement. In [32], the user association and power allocation in the mmWave based ultra dense networks (UDNs) was considered with attention to load balance constraints, user QoS requirement, energy harvesting by base stations, energy efficiency and cross-tier interference limits. In [33], the EE and SE in the SWIPT systems were investigated to achieve different EE–SE tradeoffs and optimal strategies for various constraint conditions. However, the static circuit power consumption and the harvested energy requirement have not been jointly considered in [32,33].

In this paper, firstly, we study the tradeoff between SE and EE from a green communication perspective. Moreover, we formulate two EE maximization problems subject to the constraints of minimum QoS requirements, minimum harvested energy and maximum transmit power for different practical design modes, which are two non-convex optimization problems. In particular, we aim to focus on the distributed iterative algorithm design for PS and TS modes at the receiver. To this end, the non-convex optimization problems are solved by the optimal iterative algorithms that jointly apply nonlinear fractional programming and Lagrangian dual decomposition. In addition, we consider the scenarios that the transmitter does not know or partially knows the channel state information (CSI) of the receiver to characterize the tradeoff between effective throughput (ET) and outage target rate with respect to PS and TS modes, respectively. Finally, simulation results illustrate various different interesting tradeoffs between EE and SE, between EE and the transmit power, and between EE and ET for SWIPT systems.

Compared to the previous work [19,25,26,27,28,29,30,31,32,33], the contributions of this paper are summarized as follows.

- We analyze the tradeoff between EE and SE for PS and TS modes from a green wireless communication perspective, respectively.
- We formulate the EE optimization problem as a nonlinear fractional programming problem, and propose the optimal iterative algorithms by using Dinkelbach’s method and Lagrangian dual decomposition to obtain the optimal transmit power and time switching slot.
- We analyse outage probability and give closed-form analytic expression of effective throughput under the scenarios the transmitter does not know or partially knows the CSI of receiver. The tradeoff between energy-throughput efficiency and outage target rate is analyzed for PS and TS modes, respectively.
- We characterize and analyse the corresponding performance of our proposed iterative algorithms for PS and TS modes in the SWIPT system by numerical simulations with respect to various scenarios and different condition constraints, such as minimum QoS, minimum harvested energy requirement and maximum transmit power constraint.

It is worth pointing out that energy queuing in the wireless rechargeable sensor network has been studied [6]. However, these works mainly focused on the research of energy management policy optimization at the transmitter side with energy harvesting, which is subject to random arrival energy. Therefore, it is different from our work that mainly tackles EE optimization problem for SWIPT on the receiver side.

The remainder of this paper is organized as follows. Section 2 introduces the channel model, presents the PS, TS mode for receiver architecture and gives the concept of energy efficiency of two receiver modes in the SWIPT system. Section 3 investigates the tradeoff between EE and SE. Section 4 formulates EE maximization optimization problem for the PS, TS receiver mode, respectively. Section 5 investigates the quasi-concavity of energy efficiency objective function for the PS, TS mode. Section 6 proposes distributed iterative algorithms to solve the corresponding nonlinear fractional program problem. Section 7 analyzes the tradeoffs between energy-throughput efficiency and outage target rate for PS, TS mode, respectively. Section 8 verifies the proposed iterative algorithms and the tradeoffs of SE-EE, energy throughput efficiency and outage target rate by numerical simulations. Finally, Section 9 concludes this paper.

## 2. System Model

In the WRSN system model, the energy harvesting model plays an important role in the performance of wireless power transfer (WPT). Several energy harvesting models have been investigated in the existing literature about wireless power transfer (WPT) and SWIPT [21,22,23,24,34,35,36,37,38,39,40,41,42,43,44]. Energy harvesting models are mainly divided into two categories: the Linear Energy Harvesting (EH) model [21,22,23,24] and Nonlinear Energy Harvesting (EH) model [34,35,36,37,38,39,40,41,42,43,44]. On the one hand, the linear EH model is based on the energy conversion efficiency being independent of the input power level at the wireless powered user. Thus, the total harvested energy at the energy harvesting receiver is linearly proportional to the received RF power [21,22]. On the other hand, the nonlinear EH model captures the nonlinear dynamics of the RF energy conversion efficiency for different input power levels in practical circuits and characterizes the RF energy-to-direct current (DC) power transfer at the wireless powered user terminals in the wireless energy harvesting phase, which is based on a nonlinear logistic (sigmoidal) function [35,36,37,38,39]. In fact, in [35,36,37,38,39], the authors found that the RF energy conversion efficiency will be improved as the input power increases, but for high input powers there is a diminishing return and a limitation on the maximum harvested energy. This result implies that the linear EH model be equivalent to the nonlinear model when the input power is lower, such as WRSN. Therefore, we consider employing a linear EH model in our proposed SWIPT system.

### 2.1. Channel Model

**Figure 1:**
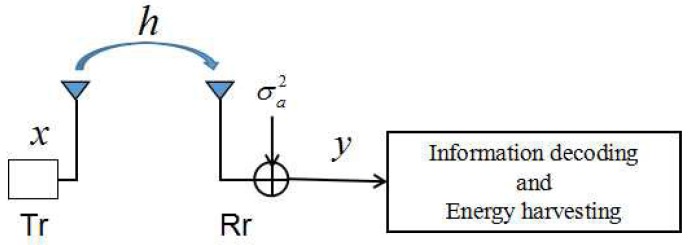
A practical transceiver design for the SWIPT system.

As shown in Figure 1, we study a point-to-point wireless link from a sensor node (SN) to cluster head (CH) with simultaneous wireless information and power transfer in a clustered WSN. It is assumed that both the SN transmitter and CH receiver are equipped with one antenna and the channel between the transmitter and the receiver is blocking fading and quasi-static. The channel model is given as follows:

(1) $$y=\sqrt{P_{t}}h_{0}x+n_{a},$$

where *x* is the transmitted signal at sensor node and *y* is the received signal at cluster head. $P_{t}$ denotes the transmit power, $h_{0}$ is the channel gain coefficient, $h=|h_{0}{|}^{2}$ is the channel power gain, $n_{a}$ represents the antenna noise and $n_{a}∼N\left(0,\sigma_{a}^{2}\right)$. Table 1 summarizes the used notations and the corresponding definitions in this paper.

### 2.2. Receiver Architecture

In this subsection, we consider the co-located receiver architecture, which means that the energy harvester and the information decoder share the same antenna so that they can observe the same channel. This architecture can be categorized into two modes, namely, power splitting (PS) mode and time switching (TS) mode. Specifically, the former means that the receiver allocates one part of power to be used for decoding information and the other for harvesting energy. The latter implies that the receiver can switch over time slots between decoding information and harvesting energy.

#### 2.2.1. Power Splitting Mode

**Figure 2:**
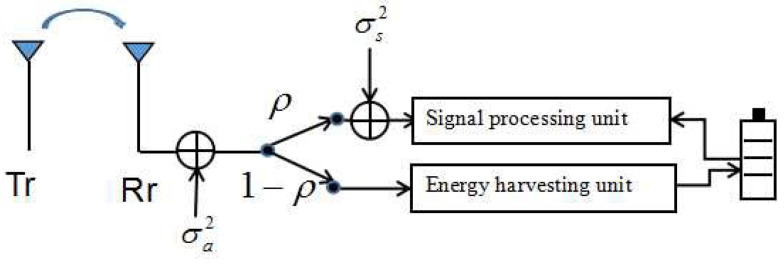
A practical transceiver design with power splitting mode.

In the power splitting mode, as shown in Figure 2, the received RF signals are split into two signal streams for the information decoder and RF energy harvester with different power levels, respectively. Let ρ∈[0,1] denote the PS coefficient for information decoding while 1−ρ is the fraction of RF signals used for energy harvesting. Therefore, the power of harvested RF signals at the energy harvester for the receiver can be given as follows:

(2) $$Q^{PS}=\xi \left(1-\rho \right)P_{t}h,$$

where ξ denotes the energy harvesting efficiency factor.

The maximum information decoding rate $R^{PS}$ at the PS receiver is given by

(3) $$R^{PS}=Wlog_{2}\left(1+\frac{\rho P_{t}h}{\rho \sigma_{s}^{2}+\sigma_{a}^{2}}\right),$$

where $\sigma_{s}^{2}$ denotes the signal processing noise power, and $\sigma_{a}^{2}$ is the antenna noise power, which is depicted in Figure 2.

#### 2.2.2. Time-Switching Mode

**Figure 3:**
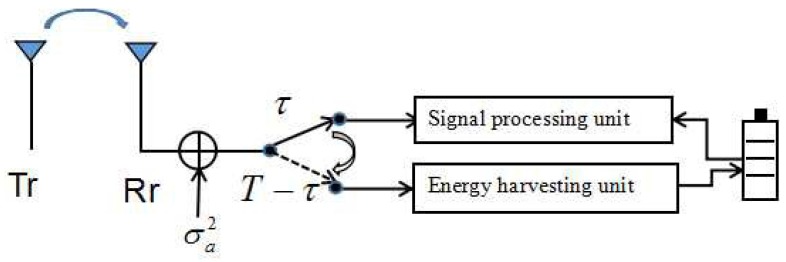
A practical transceiver design with time switching mode.

The TS mode, as shown in Figure 3, allows the receiver antenna to switch and utilize either the information decoder or the RF energy harvester for the received RF signals at a time. Hence, a time switching strategy is adopted as two phases: (i) the power transfer phase: during each coherence time interval *T*, the transmitter first transmits wirelessly the information to the receiver and then harvests energy for T−τ time slots; and (ii) the information transmission phase: the signal to the receiver for τ time slots is allocated to decode information. When the receiver works in the power transfer phase, the power harvested from the transmitter can be given as follows:

(4) $$Q^{TS}=\xi \left(T-\tau \right)P_{t}h,$$

where ξ denotes the energy harvesting efficiency factor, $P_{t}$ is the transmit power at the transmitter, and *h* denotes the channel power gain between the transmitter and the receiver. On the other hand, when the receiver works in the information decoding phase, the maximum information decoding rate $R^{TS}$ for time slot τ at the receiver is

(5) $$R^{TS}=\tau Wlog_{2}\left(1+\frac{P_{t}h}{\sigma_{a}^{2}}\right),$$

where *W* and $\sigma_{a}^{2}$ denote the fading channel bandwidth and antenna noise power, respectively.

### 2.3. Energy Efficiency

Taking both the information decode rate and the total power consumption into consideration, energy efficiency is an important metric for the SWIPT system in energy constrained wireless sensor networks. Assume that $P_{c}$ denotes the total static power consumption by mixer digital or analog converter, frequency synthesizer at the transmitter and the receiver. Note that the allocated transmission power $P_{t}$ is not concluded in the static circuit power consumption. The harvested energy $Q_{EH}$ is regarded as a compensation energy of the considered system. Then, the total power consumption of the proposed SWIPT system is formulated as

(6) $$Q_{total}=\mu P_{t}+P_{c}-Q_{EH},$$

where μ denotes the inverse of power amplification efficiency coefficient.

Then, the energy efficiency $\eta_{ee}$ of the considered system is defined as the total average number of bits successfully transmitted to the receiver per Joule energy and is given by

(7) $$\eta_{ee}=\frac{R}{Q_{total}}\left[bits/Joule\right].$$

#### 2.3.1. Power Splitting Mode

For PS mode, we assume that the transmission takes $T_{0}$ time slots, and the total consumed energy is given by

(8) $$Q_{total}^{PS}=T_{0}\left(\mu P_{t}+P_{c}-Q_{EH}^{PS}\right).$$

Then, the amount of data that can be reliably transmitted in $T_{0}$ time slots is

(9) $$R_{total}^{PS}=T_{0}\left(Wlog_{2}\left(1+\frac{\rho P_{t}h}{\rho \sigma_{s}^{2}+\sigma_{a}^{2}}\right)\right).$$

Then, according to Equations (7)–(9), the energy efficiency $\eta_{ee}^{PS}$ for PS mode can be formulated as

(10) $$\eta_{ee}^{PS}=\frac{R_{total}^{PS}}{Q_{total}^{PS}}=\frac{Wlog_{2}\left(1+\frac{\rho P_{t}h}{\rho \sigma_{a}^{2}+\sigma_{s}^{2}}\right)}{\mu P_{t}+P_{c}-\xi \left(1-\rho \right)P_{t}h}.$$

#### 2.3.2. Time Switching Mode

For TS mode, we assume that the time slot *T* is divided into two phases, i.e., power transfer phase T−τ and information decode phase τ. Therefore, the harvested energy for time slot T−τ at the receiver is given by Equation (4) and the total power consumption is presented by

(11) $$Q_{total}^{TS}=T\left(\mu P_{t}+P_{c}\right)-\xi \left(T-\tau \right)P_{t}h.$$

In time slot τ, the amount of data that can be reliably transmitted is given by Equation (5). Thus, the energy efficiency $\eta_{ee}^{TS}$ for TS mode can be formulated as

(12) $$\eta_{ee}^{TS}=\frac{R^{TS}}{Q_{total}^{TS}}=\frac{\tau Wlog_{2}\left(1+\frac{P_{t}h}{\sigma_{a}^{2}}\right)}{T\left(\mu P_{t}+P_{c}\right)-\xi \left(T-\tau \right)P_{t}h}.$$

## 3. Spectral-Energy Efficiency Tradeoff

In this section, we propose a general framework of the tradeoffs between EE and SE in the SWIPT system for different receiver modes; furthermore, we derive the specific EE–SE relation for two modes as the closed-form expression function, which are based on the proposed framework.

Traditionally, wireless sensor network design mainly aims to improve spectral efficiency and effective throughput. The spectral efficiency (SE), defined as the system throughput per unit of bandwidth, is a widely accepted criterion for wireless network optimization. However, according to Shannon capacity formulation, it becomes urgent to maintain sustainable capacity growth by only increasing transmit power. Thus, the researchers and engineers have a paradigm shift from improving system capacity and throughput to energy efficiency oriented design. Unfortunately, SE and EE of communication systems are not always consistent and sometimes conflict with each other [3]. The SE-EE tradeoff is to balance the achievable rate and energy consumption of the system for a given available bandwidth. Therefore, it is worth studying how to balance the two metrics in future systems from a green communication perspective.

In the following, we consider the static circuit power consumption $P_{c}$ in our proposed SWTPT system. On the other hand, energy harvesting terminal design is regarded as energy compensation for two modes. The significant impact on the tradeoff between SE and EE can be investigated to obtain energy-efficient transmission policies, which is crucial for environmental protection and sustainable development in WRSN.

### 3.1. Power Splitting Mode

For PS mode, let $\eta_{se}^{PS}$ denote power spectral efficiency; then we can obtain spectral efficiency expression as follows:

(13) $$\eta_{se}^{PS}=\frac{R^{PS}}{W}=log_{2}\left(1+\frac{\rho P_{t}h}{\rho \sigma_{a}^{2}+\sigma_{s}^{2}}\right).$$

Moreover, we can obtain more insight on the fundamental tradeoff between energy efficiency and spectral efficiency. According to Equation (3), the transmit power with respect to spectral efficiency is given by

(14) $$P_{t}=\frac{\left(2^{\frac{R^{PS}}{W}}-1\right)\left(\rho \sigma_{s}^{2}+\sigma_{a}^{2}\right)}{\rho h}=\frac{\left(2^{\eta_{se}^{PS}}-1\right)\left(\rho \sigma_{s}^{2}+\sigma_{a}^{2}\right)}{\rho h}.$$

Substituting Equation (14) into Equation (10), we can yield the EE as a function of the SE, i.e.,

(15) $$\eta_{ee}^{PS}=\frac{W\eta_{se}^{PS}\rho h}{\rho hP_{c}+\left(\mu -\left(1-\rho \right)\xi h\right)\left(2^{\eta_{se}^{PS}}-1\right)\left(\rho \sigma_{a}^{2}+\sigma_{s}^{2}\right)}.$$

**Figure 4:**
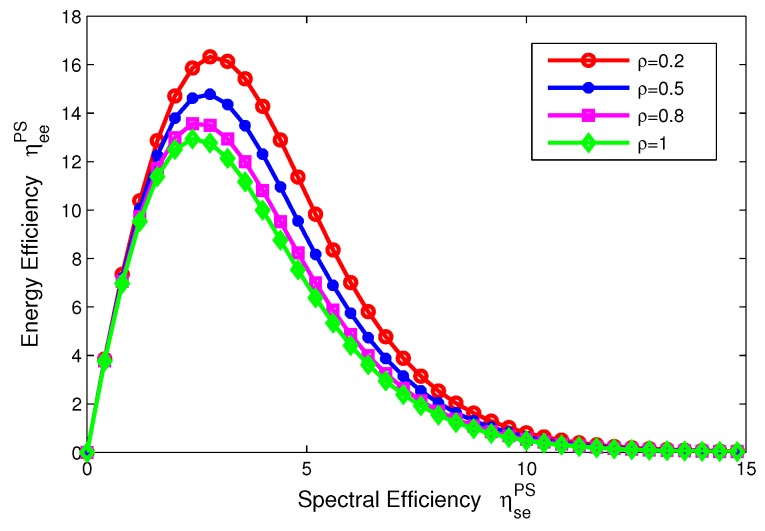
Tradeoff of EE-SE with different power splitting ratios for PS mode.

The function relation in Equation (15) is illustrated in Figure 4, which shows that the EE $\eta_{ee}^{PS}$ is quasi-concave (uni-modal) with respect to the SE $\eta_{se}^{PS}$ for PS mode, and provides more insights on the fundamental tradeoff between EE and SE in the SWIPT system.

### 3.2. Time Switching Mode

For TS mode, let $\eta_{se}^{TS}$ denote power spectral efficiency; then, the spectral efficiency for time slot τ is formulated as

(16) $$\eta_{se}^{TS}=\frac{R^{TS}}{W}=\tau log_{2}\left(1+\frac{P_{t}h}{\sigma_{a}^{2}}\right).$$

Moreover, we can obtain the transmit power as follows:

(17) $$P_{t}=\frac{\left(2^{\frac{\eta_{se}^{TS}}{\tau }}-1\right)\sigma_{a}^{2}}{h}.$$

Substituting Equation (17) into Equation (12), we can derive the relation of the SE-EE tradeoff for TS mode as follows:

(18) $$\eta_{ee}^{TS}=\frac{W\eta_{se}^{TS}h}{hTP_{c}+\left(T\mu -\left(T-\tau \right)\xi h\right)\left(2^{\frac{\eta_{se}^{TS}}{\tau }}-1\right)\sigma_{a}^{2}}.$$

**Figure 5:**
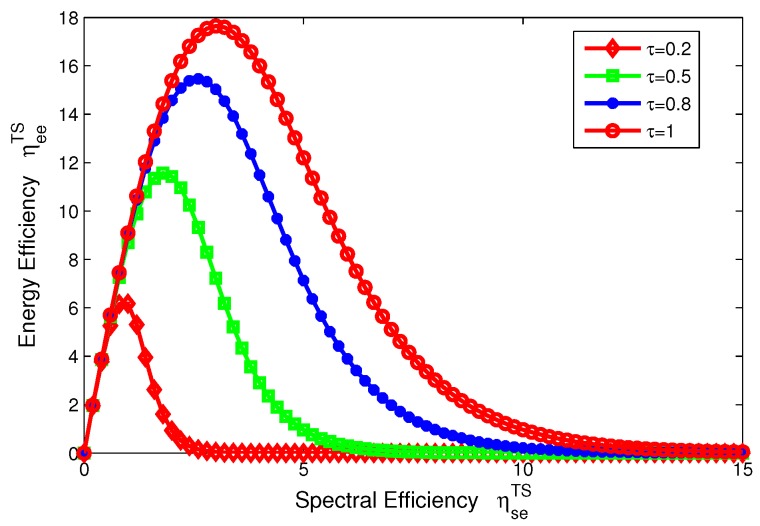
Tradeoff of EE–SE with different time switching slots for TS mode.

Similarly, the function relation in Equation (18) is illustrated in Figure 5, which shows that the EE $\eta_{ee}^{TS}$ is quasi-concave (uni-modal) with respect to the SE $\eta_{se}^{TS}$ for TS model and provides more insights on the fundamental tradeoff between EE and SE in the SWIPT system.

## 4. Problem Formulation

In this section, we first formulate the EE maximization problem for PS and TS mode, respectively. For the PS model, we propose Problem 1, constrained by minimum QoS and the minimum harvested energy requirement for optimizing transmit power and power splitting ratio. In addition, for TS mode, we formulate Problem 2 under minimum QoS constraints and minimum harvested energy requirement for optimizing transmit power and time switching slot.

### 4.1. Energy Efficiency Maximization Problem for PS Mode

For PS mode, the energy efficiency maximization problem (Problem 1) is formulated as

**Problem** **1.** (19) $$\max_{\left\{\rho ,P_{t}\right\}}\eta_{ee}^{PS},$$

*subject to*

(20) $$\begin{array}{ccc} C1 & : & R^{PS}\geq R_{min}, \end{array}$$

(21) $$\begin{array}{ccc} C2 & : & Q^{PS}\geq Q_{min}, \end{array}$$

(22) $$\begin{array}{ccc} C3 & : & 0\leq P_{t}\leq P^{max}, \end{array}$$

(23) $$\begin{array}{ccc} C4 & : & 0\leq \rho \leq 1, \end{array}$$

*where minimum rate constraint C1 indicates that the achievable rate at the receiver should be more than or equal to minimum rate requirement, i.e., minimum QoS, $R_{min}$. The minimum harvested energy constraint C2 describes that the harvested energy must be no less than the minimum energy harvesting requirement $Q_{min}$ of the wireless sensor node. C3 denotes the transmit power constraint of the transmitter, which captures the fact that the transmit power is equal to or less than the maximum power peak value of the transmitter. C4 denotes the power splitting ratio constraint. For PS mode, the feasible region for the optimization variable ρ and $P_{t}$ is given by the constraints C1–C4.*

### 4.2. Energy Efficiency Maximization Problem for TS Mode

In this subsection, for TS mode, the EE optimization problem (Problem 2) is formulated as

**Problem** **2.** (24) $$\max_{\left\{\tau ,P_{t}\right\}}\eta_{ee}^{TS},$$

*subject to*

(25) $$\begin{array}{ccc} D1 & : & R^{TS}\geq R_{min}, \end{array}$$

(26) $$\begin{array}{ccc} D2 & : & Q^{TS}\geq Q_{min}, \end{array}$$

(27) $$\begin{array}{ccc} D3 & : & 0\leq P_{t}\leq P^{max} \end{array}$$

(28) $$\begin{array}{ccc} D4 & : & 0\leq \tau \leq T, \end{array}$$

*where constraint D1 reflects that minimum transmit rate should satisfy Quality of service (QoS) requirement, D2 ensures minimum energy harvesting requirement, D3 denotes maximum transmit power constraint and D4 is the range of time switching value τ. For time switching mode, the feasible region for optimization variable τ and $P_{t}$ is characterized by the constraints D1–D4.*

It is worth noting that one of our main goals is to maximize energy efficiency under constraints C1–C4 (or D1–D4) in the SWIPT system for two modes. The EE maximization problem can be regarded as a fractional programming problem, whose objective function is the ration of two functions. It is generally a non-convex (or non-concave) function. In other words, the original fractional problem is not a convex optimization problem. In the following, we will show that the energy efficiency function is concave with respect to optimal variables while the objective function is quasi-concave.

## 5. Quasi-Concavity of Energy Efficiency Objective Function and Dinkelbach’s Method

In this section, we firstly prove the quasi-concavity of energy efficiency objective function and then, by applying Dinkelbach’s method, transform two original quasi-concave fractional programming problems into two convex optimization problems in a subtraction form. Finally, we give the distributed iteration algorithms to solve two transformed convex optimization problems by using the classical Lagrangian dual decomposition method.

### 5.1. Quasi-Concavity of Energy Efficiency Objective Function

#### 5.1.1. Power Splitting Mode

For PS mode, we give Lemmas 1 and 2 to show the quasi-concavity of the proposed EE objective function.

**Lemma** **1.** For given power splitting ratio ρ, the energy efficiency objective function $\eta_{ee}^{PS}$ in Equation *(10)* with respect to transmit power $P_{t}$ is quasi-concave.

**Proof.**  According to Equation (3), we calculate the two-order derivation of the energy efficiency function with respect to $P_{t}$ for given power splitting ratio ρ as follows:

(29) $$\frac{\partial^{2}R^{PS}}{{\left(\partial P_{t}\right)}^{2}}=\frac{W{\left(\rho h\right)}^{2}}{\ln2{\left(\rho \sigma_{a}^{2}+\sigma_{s}^{2}+\rho P_{t}h\right)}^{2}}<0.$$

Thus, the energy efficiency function $R^{PS}$ in Equation (3) with respect to the transmit power $P_{t}$ is concave.

On the other hand, the total power consumption in Equation (2) is an affine positive function with respect to the transmit power $P_{t}$. Therefore, the energy efficiency objective function $\eta_{ee}^{PS}$ is the ratio of a concave function to an affine function and results in a quasi-concave function. ☐

**Lemma** **2.** For given transmit power $P_{t}$, the energy efficiency objective function in Equation *(10)* with respect to the power splitting ratio ρ is also quasi-concave.

**Proof.**  According to Equation (3), we calculate the two-order derivation of the energy efficiency function $R^{PS}$ with respect to ρ for given transmit power $P_{t}$ as follows:

(30) $$\frac{\partial^{2}R^{PS}}{{\left(\partial \rho \right)}^{2}}=-\frac{W}{\ln2}\frac{\sigma_{s}^{2}P_{t}h\left[2\left(\rho \sigma_{a}^{4}+\rho \sigma_{a}^{2}P_{t}h+\sigma_{a}^{2}\sigma_{s}^{2}\right)+\sigma_{s}^{2}P_{t}h\right]}{{\left(\rho \sigma_{a}^{2}+\sigma_{s}^{2}+\rho P_{t}h\right)}^{2}{\left(\rho \sigma_{a}^{2}+\sigma_{s}^{2}\right)}^{2}}<0.$$

Thus, the energy efficiency function $R^{PS}$ in Equation (3) with respect to power splitting ratio ρ is concave.

On the other hand, the total power consumption is an affine positive function in Equation (10) with respect to power splitting ratio ρ. Therefore, the energy efficiency objective function $\eta_{ee}^{PS}$ is the ratio of a concave function to an affine function and results in a quasi-concave function. ☐

#### 5.1.2. Time Switching Mode

Similarly, for TS mode, the quasi-concavity of the proposed energy efficiency objective function is discussed in the proof of Lemmas 3 and 4.

**Lemma** **3.** For given time switching slot τ, the energy efficiency objective function $\eta_{ee}^{TS}$ in Equation *(12)* with respect to the transmit power $P_{t}$ is quasi-concave.

**Proof.**  According to Equation (5), we calculate the two-order derivation of the energy efficiency function $R^{TS}$ with respect to $P_{t}$ for given time switching slot τ as follows:

(31) $$\frac{\partial^{2}R^{TS}}{{\left(\partial P_{t}\right)}^{2}}=-\frac{1}{\ln2}\frac{\tau Wh^{2}}{{\left(P_{t}h+\sigma_{a}^{2}\right)}^{2}}<0.$$

Thus, the energy efficiency function $R^{TS}$ in Equation (5) with respect to the transmit power $P_{t}$ is concave.

On the other hand, the total power consumption is an affine positive function in Equation (4) with respect to the transmit power $P_{t}$. Therefore, the energy efficiency objective function $\eta_{ee}^{TS}$ is the ratio of a concave function to an affine function and results in a quasi-concave function. ☐

**Lemma** **4.** For given transmit power $P_{t}$, when D>0, the energy efficiency objective function in Equation *(12)* with respect to time switching slot τ is concave.

**Proof.**  According to Equation (12), we rewrite it with respect to variable τ as a fractional form, which is given by

(32) $$\eta_{ee}^{TS}=\frac{A\tau +B}{C\tau +D},$$

where $A=Wlog_{2}\left(1+\frac{P_{t}h}{\sigma_{a}^{2}}\right)>0$, B=0, $C=\xi P_{t}h>0$ and $D=T(\mu P_{t}+P_{c}-\xi P_{t}h)$.

We can directly calculate the two-order derivation of energy efficiency objective function $\eta_{ee}^{TS}$ with respect to τ for given transmit power $P_{t}$ as follows:

(33) $$\frac{\partial^{2}\eta_{ee}^{TS}}{{\left(\partial \tau \right)}^{2}}=-\frac{2AD}{{\left(C\tau +D\right)}^{3}}<0.$$

Therefore, the energy efficiency objective function $\eta_{ee}^{TS}$ is a concave function under the constraint D>0. ☐

**Remark** **1.** It is worth noting that the fractional programming represents a fundamental tool in energy efficiency modeling and design of wireless communication [4]. From Lemmas 1–4, we can obtain the quasi-concavity and concavity of the energy efficiency objective function, respectively. According to proposition 2.6 in [4], the quasi-concavity guarantees that the Karush–Kuhn–Tucker (KKT) conditions are necessary and sufficient for global optimality. In other words, many useful properties that hold for concave functions are still satisfied in the quasi-concave cases, such as the existence and uniqueness of the global optimal solution. Next, we transform the fractional programming into the convex optimization by using Dinkelbach’s method.

### 5.2. Dinkelbach’s Method

From the above discussion, we can know that our proposed EE maximization Problems 1 and 2 are concave-convex fractional optimization problems, which belong to a special nonlinear fractional programming and can share important properties with convex optimization theory. By using Dinklebach’s method [4], a concave-convex fractional programming can be transformed into a convex optimization problem and be solved with the aid of classical methods in convex optimization theory. In the following, we will introduce Dinklebach’s method.

Dinkelbach’s method, i.e., Dinkelbach’s algorithm, has been introduced in [4]. The basic idea is to address a concave-convex fractional problem (CCFP) by solving a sequence of easier optimization problems that converge to the global optimal solution of the CCFP. The fundamental result of Dinklebach’s algorithm is based on the relationship between the CCFP (34) and the convex function of the real optimization variable (35) with a subtract form as follows:

(34) $$\max_{x\in S}\frac{f(x)}{g(x)}$$

and

(35) $$F\left(\lambda \right)=\max_{x\in S}\left\{f\left(x\right)-\lambda g\left(x\right)\right\}$$

where f(x) is concave, differentiable, and non-negative, and g(x) is convex, differentiable, and positive. S represents compact, convex set constraints, and λ is a parameter of the auxiliary function F(λ).

In fact, the following theorem bridges the equivalent relation between CCFP (34) and (35), which is given by

**Theorem** **1.** Consider x∈S and $\lambda^{*}=\frac{f(x^{*})}{g(x^{*})}$. Then, $x^{*}$ is a solution of CCFP (*34*) if and only if

(36) $$x^{*}=argmax_{x\in S}\left\{f\left(x\right)-\lambda g\left(x\right)\right\}$$

We can observe from Theorem 1 that solving a nonlinear fractional problem is equivalent to finding the unique zero of the auxiliary function F(λ). On the other hand, we can find that Dinkelbach’s algorithm in fact follows Newton’s method as far as updating λ is concerned and presents a super-linear convergence rate in the sub-problem sequence. In a word, from an algorithm design perspective, Dinkelbach’s algorithm is an iterative algorithm to find the increasing values of feasible λ by solving the parameterized optimization problem of $\max_{x\in S}F\left(\lambda_{n}\right)=\max_{x\in S}\left\{f\left(x\right)-\lambda_{n}g\left(x\right)\right\}$ at the *n*-th iteration. The iterative process stops until $\left|F(\lambda_{n})\right|$ is less than or equal to a pre-determined tolerance value ε, which is described in Algorithm 1.

**Algorithm 1** Dinkelbach’s method1:Initialize: ε>0, n=0;2:Set $\lambda_{n}=0$3:**while** $|F(\lambda_{n})|>\varepsilon$ **do**4: $x^{*}=argmax_{x\in S}\left\{f\left(x\right)-\lambda_{n}g\left(x\right)\right\}$;5: $F\left(\lambda_{n}\right)=f\left(x_{n}^{*}\right)-\lambda_{n}g\left(x_{n}^{*}\right)$6: $\lambda_{n+1}=\frac{f(x_{n}^{*})}{g(x_{n}^{*})}$7: n=n+18:**end while**


## 6. The Proposed Algorithm for Solving Transformed Optimization Problems

In this section, we mainly focus on solving the proposed optimization problems by applying Dinkelbach’s method and Lagrangian dual decomposition. Dinkelbach’s method for EE maximization for PS and TS mode is described in Algorithm 2.

**Algorithm 2** Dinkelbach’s method for EE maximization1:Input: $L_{\max}^{outer}:$ the maximum number of iterations and $\epsilon^{outer}:$ the maximum tolerance;2:$\eta_{0}\leftarrow 0$;3:i←0;4:**while** $k\leq L_{\max}^{outer}$ and $|\eta_{i}-\eta_{i-1}|>\varepsilon^{outer}$ **do**5: i←i+1;6: Obtain the optimal transmit power ${P_{t}}^{*}$ and the power splitting ratio $\rho^{*}$ (inner loop) by solving Problem 3;7: Obtain the optimal transmit power ${P_{t}}^{*}$ and the time switching slot $\tau^{*}$ (inner loop) by solving Problem 4;8: Update $\eta_{i}\leftarrow \frac{R^{PS}\left(P_{t}^{*},\rho^{*}\right)}{Q^{PS}\left(P_{t}^{*},\rho^{*}\right)}$ for PS mode;9: Update $\eta_{i}\leftarrow \frac{R^{TS}\left(P_{t}^{*},\tau^{*}\right)}{Q^{TS}\left(P_{t}^{*},\tau^{*}\right)}$ for TS mode;10:**end while**11:**return**


### 6.1. Power Splitting Mode

For PS mode, according to the formulated fractional Problem 1 and applying Dinkelbach’s method discussed in above section, the parametric version of the EE-maximization Problem 1 is reformulated as

**Problem** **3.** (37) $$\max_{\left\{\rho ,P_{t}\right\}}\left\{R^{PS}-\eta_{ee}^{PS}Q^{PS}\right\},$$

(38) s.t.C1∼C4.

Here, $R^{PS}$ and $Q^{PS}$ with respect to optimization variable $\rho ,P_{t}$ are concave and affine, respectively. Then, the objective function is concave in Problem 3 and the feasible region generated by constraints C1–C4 is a convex set. Thus, Problem 3 is a convex optimization problem. Furthermore, we can obtain the primal solution with a zero duality gap by solving a dual problem. In the following, the Lagrangian function over variable $\rho ,P_{t}$ for optimization Problem 3 is presented by

(39) $$L\left\{\rho ,P_{t},\varphi ,\phi ,\psi \right\}=R^{PS}-\eta_{ee}^{PS}Q^{PS}+\varphi \left(R^{PS}-R_{min}\right)+\phi \left(Q^{PS}-Q_{min}\right)+\psi \left(P^{max}-P_{t}\right),$$

where φ,ϕ,ψ denote the Lagrange multiplier corresponding to the constraints C1, C2 and C3, respectively.

Since Problem 3 is a standard form of the convex optimization problem, we can deal with the updating process of the primal and dual variables in terms of the Karush–Kuhn–Tucker (**KKT**) first order optimality conditions in [45], in order to find the optimal solution. In the following, we mainly focus on updating optimization variables and Lagrangian multiplier to obtain the solution for PS modes.

#### 6.1.1. Optimal Transmit Power for PS Mode

For given power splitting ratio ρ, we can obtain the first order partial derivation of Lagrangian function with respect to transmit power $P_{t}$ and let it equal zero as follows:

(40) $$\frac{\partial L}{\partial P_{t}}=\frac{W\rho h(1+\varphi )}{\ln2(\rho P_{t}h+\rho \sigma_{a}^{2}+\sigma_{s}^{2})}+\eta_{ee}^{PS}-\psi +\left(\phi -\eta_{ee}^{PS}\right)\xi \left(1-\rho \right)h=0.$$

By simple calculation, optimal transmit power $P_{t}^{*}$ is given by

(41) $$P_{t}^{*}={\left\{\frac{W(1+\varphi )}{\ln2[\left(\psi -\eta_{ee}^{PS}\mu \right)+\left(\eta_{ee}^{PS}\mu -\phi \right)\xi \left(1-\rho \right)h]}-\frac{\rho \sigma_{a}^{2}+\sigma_{s}^{2}}{\rho h}\right\}}_{0}^{P^{max}},$$

where ${\left\{\Theta \right\}}_{0}^{P^{max}}$ denotes $0\leq \Theta \leq P^{max}$.

#### 6.1.2. Optimal Power Splitting Ratio

On the other hand, for fixed transmit power $P_{t}$, we can give the first order derivation of Lagrangian function with respect to variable ρ as follows:

(42) $$\frac{\partial L}{\partial \rho }=\frac{W(1+\varphi )}{\ln2}\left[\frac{\sigma_{a}^{2}+P_{t}h}{\rho \sigma_{a}^{2}+\sigma_{s}^{2}+\rho P_{t}h}-\frac{\sigma_{a}^{2}}{\rho \sigma_{a}^{2}+\sigma_{s}^{2}}\right]+\left(\eta_{ee}^{PS}-\phi \right)\xi P_{t}h=0.$$

By solving Equation (42), the optimal power splitting ratio $\rho^{*}$ is given by

(43) $$\rho^{*}={\left\{\frac{\sigma_{s}^{2}\left[-M_{0}+\sqrt{M_{0}^{2}-4\left(M_{0}-\sigma_{a}^{2}\right)\left(\sigma_{s}^{2}-M\right)}\right]}{2\left(M_{0}-\sigma_{a}^{2}\right)\sigma_{a}^{2}}\right\}}_{0}^{1},$$

where $M=\frac{W(1+\varphi )}{\ln2[\xi \left(\phi -\eta_{ee}^{PS}\right)]}$, $M_{0}=2\sigma_{a}^{2}+P_{t}h$ and ${\left\{\Theta \right\}}_{0}^{1}$ denotes 0≤Θ≤1.

#### 6.1.3. Lagrange Multiplier Update for PS Mode

In the following, we apply the sub-gradient method to update the dual variables φ, ϕ and ψ:

(44) $$\begin{array}{ccc} \varphi \left(k+1\right) & = & {\left[\varphi \left(k\right)+\alpha_{1}\left(R^{PS}-R_{min}\right)\right]}^{+}, \end{array}$$

(45) $$\begin{array}{ccc} \phi \left(k+1\right) & = & {\left[\phi \left(k\right)+\alpha_{2}\left(Q^{PS}-Q_{min}\right)\right]}^{+}, \end{array}$$

(46) $$\begin{array}{ccc} \psi \left(k+1\right) & = & {\left[\psi \left(k\right)+\alpha_{3}\left(P^{max}-P_{t}\right)\right]}^{+}, \end{array}$$

where $\alpha_{1}$, $\alpha_{2}$ and $\alpha_{3}$ denote the proper step size of sub-gradient iteration, respectively, and ${\left[\Theta \right]}^{+}$ denotes max{0,Θ}. For PS mode, the inner loop iterative algorithm for obtaining the optimal transmit power ${P_{t}}^{*}$ and the power splitting ratio $\rho^{*}$ for given $\eta_{ee}^{PS}$ is illustrated in Algorithm 3, respectively.

**Algorithm 3** Inner Loop Iterative Algorithm for obtaining ${P_{t}}^{*}$ and $\rho^{*}$ for given $\eta_{ee}^{PS}$1:Input: $L_{\max}^{inner}:$ the maximum number of iterations and $\epsilon^{inner}:$ the maximum tolerance;2:k←0;3:**while** $k\leq L_{\max}^{inner}$ or $|\varphi \left(k+1\right)-\varphi \left(k\right)|<\varepsilon^{inner}$ and $|\phi \left(k+1\right)-\phi \left(k\right)|<\varepsilon^{inner}$ and $|\psi \left(k+1\right)-\psi \left(k\right)|<\varepsilon^{inner}$ **do**4: k+1←k;5: Obtain the optimal transmit power ${P_{t}}^{*}$ by using Equation (41);6: Obtain the power splitting ratio $\rho^{*}$ by using Equation (43);7: Update the dual variables φ,ϕ,ψ by using Equations (44)–(46);8:**end while**9:**return**


### 6.2. Time Switching Mode

For TS mode, similarly, according to the formulated fractional program Problem 2, the EE-maximization Problem 2 is reformulated as

**Problem** **4.** (47) $$\max_{\left\{\tau ,P_{t}\right\}}\left\{R^{TS}-\eta_{ee}^{TS}Q^{TS}\right\},$$

(48) s.t.D1∼D4,

Since $R^{TS}$ and $Q^{TS}$ with respect to optimization variable $P_{t}$ are concave and affine, respectively. In addition, the objective function is concave in Problem 4 and the feasible region generated by constraints D1∼D4 is a convex set. Therefore, Problem 4 is a convex optimization problem. Furthermore, the Lagrangian function over variable $P_{t}$ for optimization Problem 4 is described by

(49) $$L_{0}\left\{\tau ,P_{t},\varphi_{0},\phi_{0},\psi_{0}\right\}=R^{TS}-\eta_{ee}^{TS}Q^{TS}+\varphi_{0}\left(R^{TS}-R_{min}\right)+\phi_{0}\left(Q^{TS}-Q_{min}\right)+\psi_{0}\left(P^{max}-P_{t}\right),$$

where $\varphi_{0},\phi_{0},\psi_{0}$ are the corresponding Lagrangian multipliers of constraint conditions.

Since Problem 1 is convex, we can give an iterative update of the primal and dual variables in terms of the Karush–Kuhn–Tucker (KKT) first order optimality conditions of [45], which is to search the global optimal solution. Moreover, we update optimization variables and Lagrange multipliers to obtain the optimal solution for TS mode.

#### 6.2.1. Optimal Transmit Power for TS Mode

For given switching time τ, we can calculate the first order partial derivation of Lagrangian function with respect to transmit power $P_{t}$ and let it equal zero, i.e.,

(50) $$\frac{\partial L_{0}}{\partial P_{t}}=\frac{\tau Wh(1+\varphi_{0})}{\ln2(\sigma_{a}^{2}+P_{t}h)}+\eta_{ee}^{TS}\left(T\mu +\xi \left(T-\tau \right)h\right)-\psi_{0}=0.$$

By simple calculation, optimal transmit power $P_{t}^{*}$ is given by

(51) $$P_{t}^{*}={\left\{\frac{\tau W(1+\varphi_{0})}{\ln2[\psi_{0}-\eta_{ee}^{ST}\left(T\mu +\xi \left(T-\tau \right)h\right)]}-\frac{\sigma_{a}^{2}}{h}\right\}}_{0}^{P^{max}},$$

where ${\left\{\Theta \right\}}_{0}^{P^{max}}$ denotes $0\leq \Theta \leq P^{max}$.

#### 6.2.2. Optimal Time Switching Slot for TS Mode

On the other hand, according to Lemma 4, we know that the objective function $\eta_{ee}^{TS}$ with respect to time switching variable τ is concave. Thus, the Lagrangian function can be reformulated as

(52) $$L_{1}\left\{\tau ,P_{t},\varphi_{1},\phi_{1},\psi_{1},\nu_{1}\right\}=\eta_{ee}^{TS}+\varphi_{1}\left(R^{TS}-R_{min}\right)+\phi_{1}\left(Q^{TS}-Q_{min}\right)+\psi_{1}\left(P^{max}-P_{t}\right)+\nu_{1}\left(T-\tau \right).$$

In the following, for fixed transmit power $P_{t}$, we can give the first order derivation of of Lagrangian function (52) with respect to time switching slot variable τ and let it equal zero as follows:

(53) $$\frac{\partial L_{1}}{\partial \tau }=\frac{AD}{{\left(C\tau +D\right)}^{2}}+\varphi_{1}A-\phi_{1}\xi P_{t}h-\nu_{1}=0.$$

By solving the equation, the optimal time switching slot $\tau^{*}$ is given by

(54) $$\tau^{*}={\left\{\frac{1}{C}\sqrt{\frac{AD}{\phi_{1}\xi P_{t}h-\varphi_{1}A+\nu_{1}}}-\frac{D}{C}\right\}}_{0}^{T},$$

where $A=Wlog_{2}\left(1+\frac{P_{t}h}{\sigma_{a}^{2}}\right)>0$, $C=\xi P_{t}h>0$, $D=T(\mu P_{t}+P_{c}-\xi P_{t}h)$ and ${\left\{\Theta \right\}}_{0}^{T}$ denotes 0≤Θ≤T.

#### 6.2.3. Lagrange Multiplier Update for TS Mode

In this subsection, we apply the sub-gradient algorithm to update the dual variables $\varphi_{0}$, $\phi_{0}$, $\psi_{0}$ as follows:

(55) $$\begin{array}{ccc} \varphi_{0}\left(k+1\right) & = & {\left[\varphi_{0}\left(k\right)+\beta_{1}\left(R^{TS}-R_{min}\right)\right]}^{+}, \end{array}$$

(56) $$\begin{array}{ccc} \phi_{0}\left(k+1\right) & = & {\left[\phi_{0}\left(k\right)+\beta_{2}\left(Q^{TS}-Q_{min}\right)\right]}^{+}, \end{array}$$

(57) $$\begin{array}{ccc} \psi_{0}\left(k+1\right) & = & {\left[\psi_{0}\left(k\right)+\beta_{3}\left(P^{max}-P_{t}\right)\right]}^{+}, \end{array}$$

where $\beta_{1}$, $\beta_{2}$, $\beta_{3}$ denote the proper iteration step size of sub-gradient algorithm, respectively, and ${\left[\Theta \right]}^{+}$ denotes max{0,Θ}.

Next, we also apply the sub-gradient algorithm to update the dual variables $\varphi_{1}$, $\phi_{1}$, $\psi_{1}$ and $\nu_{1}$ as follows:

(58) $$\begin{array}{ccc} \varphi_{1}\left(k+1\right) & = & {\left[\varphi_{1}\left(k\right)+\gamma_{1}\left(R^{TS}-R_{min}\right)\right]}^{+}, \end{array}$$

(59) $$\begin{array}{ccc} \phi_{1}\left(k+1\right) & = & {\left[\phi_{1}\left(k\right)+\gamma_{2}\left(Q^{TS}-Q_{min}\right)\right]}^{+}, \end{array}$$

(60) $$\begin{array}{ccc} \psi_{1}\left(k+1\right) & = & {\left[\psi_{1}\left(k\right)+\gamma_{3}\left(P^{max}-P_{t}\right)\right]}^{+}, \end{array}$$

(61) $$\begin{array}{ccc} \nu_{1}\left(k+1\right) & = & {\left[\nu_{1}\left(k\right)+\gamma_{4}\left(T-\tau \right)\right]}^{+}, \end{array}$$

where $\gamma_{1}$, $\gamma_{2}$, $\gamma_{3}$, $\gamma_{4}$ denote the proper step size of sub-gradient algorithm, and ${\left[\Theta \right]}^{+}$ denotes max{0,Θ}. For TS mode, an inner loop iterative algorithm for obtaining the optimal transmit power ${P_{t}}^{*}$ and the time switching slot $\tau^{*}$ for given $\eta_{ee}^{TS}$ is illustrated in the following Algorithms 4 and 5, respectively.

**Algorithm 4** Inner Loop Iterative Algorithm for obtaining ${P_{t}}^{*}$ for given $\eta_{ee}^{TS}$1:Input: $L_{\max}^{inner}:$ the maximum number of iterations and $\epsilon^{inner}:$ the maximum tolerance;2:k←0;3:**while** $k\leq L_{\max}^{inner}$ or $|\varphi_{0}\left(k+1\right)-\varphi_{0}\left(k\right)|<\varepsilon^{inner}$ and $|\phi_{0}\left(k+1\right)-\phi_{0}\left(k\right)|<\varepsilon^{inner}$ and $|\psi_{0}\left(k+1\right)-\psi_{0}\left(k\right)|<\varepsilon^{inner}$ **do**4:k+1←k;5: Obtain the optimal transmit power ${P_{t}}^{*}$ by using Equation (51);6: Update the dual variables $\varphi_{0},\phi_{0},\psi_{0}$ by using Equations (55)–(57);7:**end while**8:**return**


**Algorithm 5** Inner Loop Iterative Algorithm for obtaining $\tau^{*}$ for given $\eta_{ee}^{TS}$1:Input: $L_{\max}^{inner}:$ the maximum number of iterations and $\epsilon^{inner}:$ the maximum tolerance;2:k←0;3:**while** $k\leq L_{\max}^{inner}$ or $|\varphi_{1}\left(k+1\right)-\varphi_{1}\left(k\right)|<\varepsilon^{inner}$ and $|\phi_{1}\left(k+1\right)-\phi_{1}\left(k\right)|<\varepsilon^{inner}$ and $|\psi_{1}\left(k+1\right)-\psi_{1}\left(k\right)|<\varepsilon^{inner}$ and $|\nu_{1}\left(k+1\right)-\nu_{1}\left(k\right)|<\varepsilon^{inner}$ **do**4:k+1←k;5: Obtain the optimal time switching slot $\tau^{*}$ by using Equation (54);6: Update the dual variables $\varphi_{1},\phi_{1},\psi_{1},\nu_{1}$ by using Equations (58)–(61);7:**end while**8:**return**


### 6.3. Computational Complexity Analysis

In this section, we analyze computational complexity of our proposed algorithm as given by Algorithms 3–5 in the SWIPT system.

Our proposed iterative algorithm is divided into two-layer iterative loops. Specifically, the inner main loop is to solve Problem 3 (or Problem 4) for a given parameter $\eta_{ee}^{PS}$ (or $\eta_{ee}^{TS}$) by the dual decomposition method. Next, the parameter $\eta_{ee}^{PS}$ (or $\eta_{ee}^{TS}$) is updated for solving Problem 3 (or Problem 4) in the next iteration. Since the proposed algorithm can converge to the the optimal solution of Problem 3 (or Problem 4), this procedure will repeat until convergence is achieved or the number of iterations reaches $L_{max}$ and the maximum tolerance satisfies $\varepsilon <10^{-5}$. Since the gradient method is adopted to update the Lagrange multiplier, then the time complexity of outer layer loop is sup-linear, i.e., $O(L_{max}^{outer})$. On the other hand, Problem 3 (or Problem 4) is convex with respect to the optimization variables. In other words, solving the inner loop optimization problem by Dinkelbach’s method needs a polynomial time complexity, i.e., $O(L_{max}^{inner})$. In summary, our proposed algorithm has a polynomial time complexity, i.e., $O(L_{max}^{outer}\times L_{max}^{inner})$, which is desirable to apply in the practical SWIPT system.

## 7. Effective Throughput and Energy-Throughput Efficiency

In this section, we consider the scenario that the transmitter does not know or partly knows the channel state information (CSI) of the receiver. Therefore, we investigate the effective throughput of link between the transmitter and the receiver by using outage target rate and outage probability. Assume that $R_{0}^{PS}$ and $R_{0}^{TS}$ represent the critical value of reliable transmission outage occurrence for PS, TS mode, respectively. Moreover, we derive the close-form expression of effective throughput for two modes. Furthermore, we define energy-throughput efficiency and characterize the relationship between energy-throughput efficiency and the outage target rate. Finally, we derive the optimal value of outage target rate, which is regarded as a system parameter to maximize the energy-throughput efficiency.

We assume that the channel power gain *h* satisfies the exponential distribution. The probability density function (PDF) of *h* is given by

(62) $$f_{h}\left(z\right)=\frac{1}{\bar{h}}exp\left(-\frac{z}{\bar{h}}\right),$$

where $\bar{h}$ denotes the expectation of *h*. Then, the cumulative density functions (CDF) of *h* is given by

(63) $$F_{h}\left(z\right)=1-exp\left(-\frac{z}{\bar{h}}\right).$$

In the following, we derive the closed form expression of outage probability and effective throughput, and then give the definition of energy-throughput efficiency for two modes.

### 7.1. Power Splitting Mode

For PS mode, the outage probability of reliable transmission is defined as the probability that the reliable transmission rate is less than the outage target rate, which is obtained by

(64) $$\begin{array}{cc} P_{out}^{PS}\left(\rho ,P_{t},R_{0}^{PS}\right) & =Pr\{R^{PS}\left(\rho ,P_{t}\right)<R_{0}^{PS}\}\\ & =Pr\{h<\frac{\left(2^{\frac{R_{0}^{PS}}{W}}-1\right)\left(\rho \sigma_{a}^{2}+\sigma_{s}^{2}\right)}{\rho P_{t}}\}\\ & =1-exp(-\frac{\left(2^{\frac{R_{0}^{PS}}{W}}-1\right)\left(\rho \sigma_{a}^{2}+\sigma_{s}^{2}\right)}{\bar{h}\rho P_{t}}). \end{array}$$

Moreover, the effective throughput of reliable transmission for PS mode is defined as the product between the outage target rate and the probability of success reliable transmission, which is given by

(65) $$T^{PS}\left(\rho ,P_{t},R_{0}^{PS}\right)=R_{0}^{PS}\left(1-P_{out}^{PS}\left(\rho ,P_{t},R_{0}^{PS}\right)\right)=R_{0}^{PS}exp\left(-\frac{\left(2^{\frac{R_{0}^{PS}}{W}}-1\right)\left(\rho \sigma_{a}^{2}+\sigma_{s}^{2}\right)}{\bar{h}\rho P_{t}}\right).$$

Finally, according to the previous definition, the energy-throughput efficiency of the SWIPT system for power splitting mode on effective throughput is defined as the ratio between the effective throughput and the total consumed energy, which can be expressed as

(66) $$\eta_{ee}\left(\rho ,P_{t},R_{0}^{PS}\right)=\frac{T^{PS}\left(\rho ,P_{t},R_{0}^{PS}\right)}{Q_{total}}.$$

Next, for given $\rho ,P_{t}$, we characterize the relationship between energy-throughput efficiency $\eta_{ee}\left(\rho ,P_{t},R_{0}^{PS}\right)$ and outage target rate $R_{0}^{PS}$. We observe that the energy throughput efficiency with respect to outage target rate is quasi-concave. In fact, we can obtain the following expression by taking natural logarithm at both sides of Equation (66):

(67) $$\ln\eta_{ee}\left(\rho ,P_{t},R_{0}^{PS}\right)=\ln R_{0}^{PS}+\left(-\frac{\left(2^{\frac{R_{0}^{PS}}{W}}-1\right)\left(\rho \sigma_{a}^{2}+\sigma_{s}^{2}\right)}{\bar{h}\rho P_{t}}\right)-\ln Q_{total}.$$

We can observe that $\ln\eta_{ee}\left(\rho ,P_{t},R_{0}^{PS}\right)$ is a strict concave function with respect to $R_{0}^{PS}$ from the right-hand side of the equality (67). Thus, $\eta_{ee}\left(\rho ,P_{t},R_{0}^{PS}\right)$ is a strict log-concave function with respect to $R_{0}^{PS}$. Furthermore, it is also a strict quasi-concave function with respect to $R_{0}^{PS}$.

Then, for given ρ and $P_{t}$, the optimal value of outage target rate exists and can be regarded as a system parameter to maximize energy-throughput efficiency, i.e.,

(68) $$R_{0}^{PS*}=argmax_{\left\{\rho ,P_{t}\right\}}\eta_{ee}\left(\rho ,P_{t},R_{0}^{PS}\right).$$

### 7.2. Time Switch Mode

On the other hand, we can obtain the outage probability, effective throughput and energy throughput efficiency for time switching mode as follows:

(69) $$\begin{array}{cc} P_{out}^{TS}\left(\tau ,P_{t},R_{0}^{TS}\right) & =Pr\{R^{TS}\left(\tau ,P_{t}\right)<R_{0}^{TS}\}\\ & =Pr\{h<\frac{\left(2^{\frac{R_{0}^{TS}}{\tau W}}-1\right)\sigma_{a}^{2}}{P_{t}}\}\\ & =1-exp(-\frac{\left(2^{\frac{R_{0}^{TS}}{\tau W}}-1\right)\sigma_{a}^{2}}{\bar{h}P_{t}}). \end{array}$$

Moreover, the effective throughput of reliable transmission for TS mode is given by

(70) $$T^{TS}\left(\tau ,P_{t},R_{0}^{TS}\right)=R_{0}^{TS}exp\left(-\frac{\left(2^{\frac{R_{0}^{TS}}{\tau W}}-1\right)\sigma_{a}^{2}}{\bar{h}P_{t}}\right).$$

Similarly, the energy-throughput efficiency of the SWIPT system with time switching mode on effective throughput can be given by

(71) $$\eta_{ee}\left(\tau ,P_{t},R_{0}^{TS}\right)=\frac{T^{TS}\left(\tau ,P_{t},R_{0}^{TS}\right)}{Q_{total}}.$$

For TS mode, similar to PS mode, we also can prove that the energy-throughput efficiency $\eta_{ee}\left(\tau ,P_{t},R_{0}^{TS}\right)$ with respect to outage target rate $R_{0}^{TS}$ is quasi-concave. Then, for given τ and $P_{t}$, the optimal value of outage target rate exists and can be obtained by

(72) $$R_{0}^{TS*}=argmax_{\left\{\tau ,P_{t}\right\}}\eta_{ee}\left(\tau ,P_{t},R_{0}^{TS}\right).$$

## 8. Numerical Simulation Results and Discussion

In this section, we first verify the convergence of the proposed algorithm in Section 5. Moreover, we compare and evaluate the performance of our optimal solution for different parameters, such as the transmit power $P_{t}$, power splitting ratio ρ and time switching slot τ for PS,TS mode, respectively. In addition, we characterize the effects of minimum QoS and minimum harvested energy requirement on energy efficiency. Furthermore, we illustrate the tradeoffs between EE and SE and characterize the quasi-concavity of the EE with respect to SE for PS, TS mode, respectively. Finally, we illustrate the quasi-concavity of the energy-throughput efficiency with respect to the outage target rate and evaluate the optimal value of outage target rate, which maximizes the energy-throughput efficiency for two modes, respectively.

We consider a SISO SWIPT system with PS, TS mode and the used simulation parameter values for SWIPT system are presented in Table 2. We assume that the total bandwidth of fading channel is 100 MHz. Moreover, the static circuit power consumption $P_{c}$ is 10 mW, which is considered as a constant parameter. Furthermore, unless specified otherwise, we assume that the system satisfies a minimum rate requirement of $R_{min}$ = 1 kbps, minimum harvested energy constraint of $Q_{min}=$ 0.1 J, and initial energy harvesting efficiency ξ=1, the power amplifier coefficient μ=2. For the sake of fast convergence, we assume that the step size of Lagrangian multiplier update $\alpha_{i}\left(i=1,2,3\right)$, $\beta_{j}\left(j=1,2,3\right)$, $\gamma_{l}\left(l=1,2,3,4\right)=-0.05$, convergence tolerance of iterative algorithms $\varepsilon_{outer}^{D}=\varepsilon_{inner}^{D}=10^{-5}$, maximum number of inner loop iterations $L_{inner}^{D}=100$ and maximum number of outer loop iterations $L_{outer}^{D}=20$.

### 8.1. The Tradeoffs of Energy-Spectral Efficiency

In this subsection, we characterize the tradeoffs between energy efficiency and spectral efficiency for two modes in the SWIPT system, which are depicted in Figure 4 and Figure 5. If only transmit power is considered, we can see that the EE decreases as the SE increases and the EE-SE relation looks contradictory. However, in a practical communication system, in addition to the transmit power, there exist other kinds of power consumed to maintain the whole system, such as the static circuit power $P_{c}$. Then, the circuit power consumption is considered as a constant power for two transceivers in our proposed SWIPT system and more details can be found in Section 3.

For the PS mode, the EE–SE relation presented in Equation (15) is showed in Figure 4. For comparison, the power splitting ratio ρ is set to 0.2, 0.5, 0.8, 1. In Figure 4, we can see that an optimal peak value of the EE can be achieved in different levels of the power splitting ratio. In other words, the EE-SE relation does not conflict any more and the optimal tradeoff can be achieved. On the other hand, Figure 4 shows that the EE decreases as the power splitting ratio increases. In particular, ρ=1 means no energy harvesting, which indicates that the SWIPT system can enhance energy efficiency under the condition of the same spectral efficiency for PS mode.

For the TS mode, the EE-SE relation presented in Equation (18) is showed in Figure 5. For comparison, the time switching slot τ is set to 0.2, 0.5, 0.8, 1. In Figure 5, an optimal value of tradeoff between EE and SE can be obtained in different intervals of the time switching slot. Similarly, the EE–SE relation is not contradictory any more and the optimal tradeoff can be achieved. In addition, Figure 5 illustrates that the EE increases as the time switching slot increases as well as the spectral efficiency, which shows that the SWIPT system can achieve different EE–SE tradeoff levels by adjusting time switching slot τ for TS mode.

### 8.2. Convergence of Iterative Algorithms

**Figure 6:**
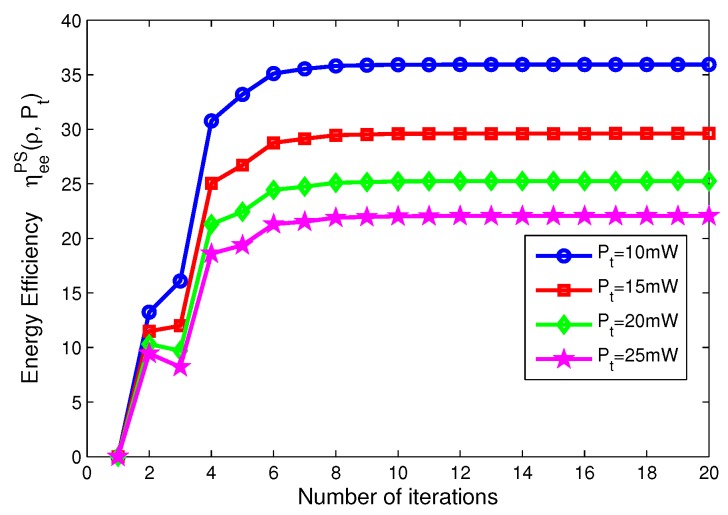
Energy efficiency versus number of iterations with different transmit power for PS mode.

**Figure 7:**
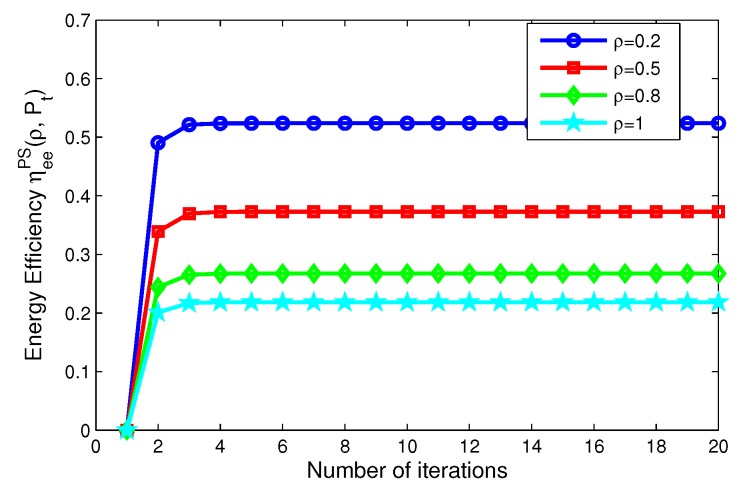
Energy efficiency versus number of iterations with different power splitting ratios for PS mode.

**Figure 8:**
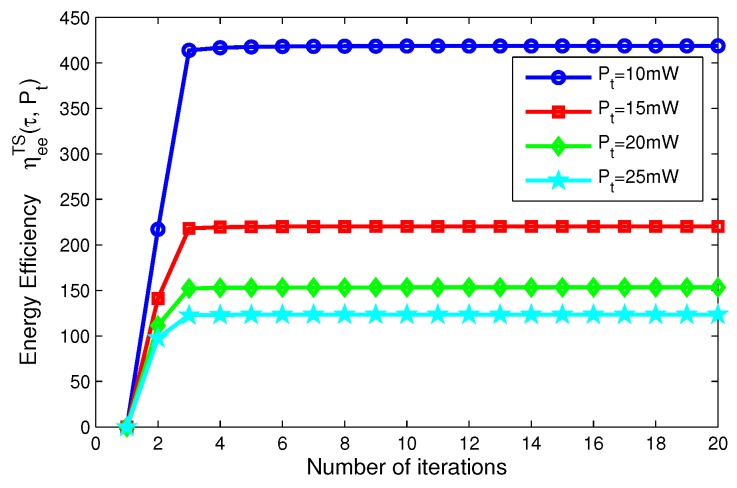
Energy efficiency versus number of iterations with different transmit power for TS mode.

**Figure 9:**
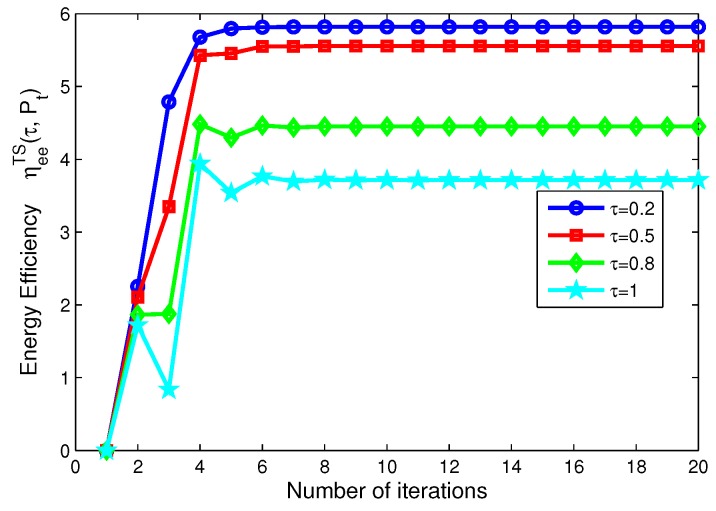
Energy efficiency versus number of iterations with different time switching slots for TS mode.

In this subsection, we focus on the energy efficiency versus the number of iterations and the convergence speed of the our proposed iterative algorithms for PS, TS mode, which are depicted in Figure 6, Figure 7, Figure 8 and Figure 9. Specifically, for PS mode, Figure 6 depicts the energy efficiency of the proposed iterative algorithms for different levels of transmit power versus the number of iterations. Figure 6 reveals that the larger the transmit power is, the lower the energy efficiency of system is, when $P_{t}=10,15,20,25$ mW. Figure 7 shows the energy efficiency of the proposed algorithms for different power splitting ratios versus number of iterations. Figure 7 illustrates that the higher the power splitting ratio is, the lower the energy efficiency of system is, when ρ= 0.2, 0.5, 0.8, 1, respectively.

On the other hand, for TS mode, Figure 8 depicts the energy efficiency of the proposed algorithms for different transmit power versus the number of iterations. Figure 8 reveals that the larger the transmit power is, the lower the energy efficiency of system is, when $P_{t}=10,\;15,\;20,\;25$ mW, respectively. Figure 9 shows the energy efficiency of the proposed algorithms for different time switching slots versus the number of iterations. Figure 9 illustrates that the higher the time switching slot is, the lower the energy efficiency of system is, when τ=0.2, 0.5, 0.8, 1, respectively.

In addition, in Figure 6, Figure 7, Figure 8 and Figure 9, after only eight iterations, the proposed iterative algorithms achieve convergence for all considered scenarios. In addition, the convergence speed of the proposed algorithms is unchanged for different transmit power, power splitting ratio and time switching slots, which is expected for the practical SWIPT system.

### 8.3. Effects of Minimum QoS and Minimum Harvested Energy on Energy Efficiency for PS Mode

**Figure 10:**
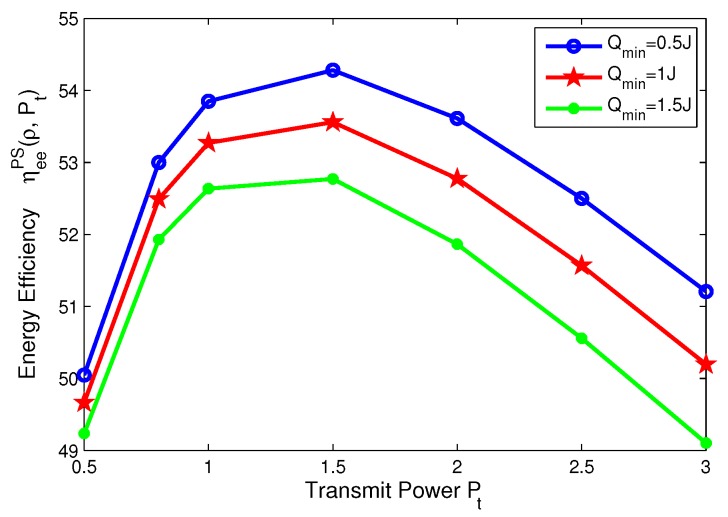
Energy efficiency versus transmit power with different minimum harvested energy requirements for PS mode.

**Figure 11:**
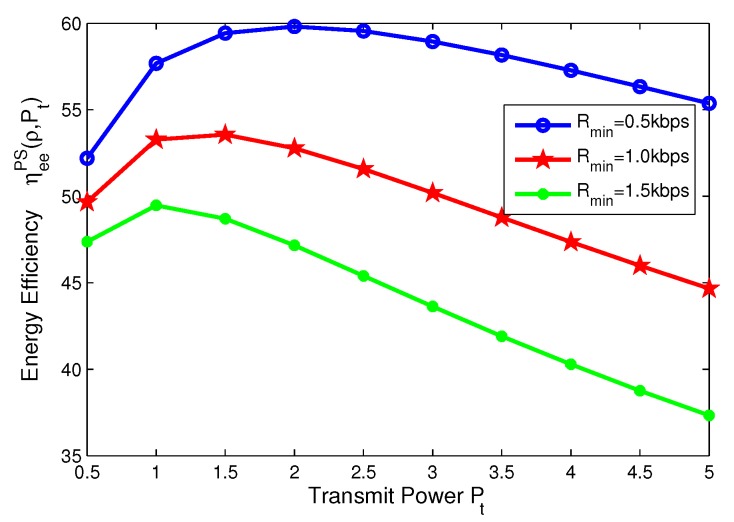
Energy efficiency versus transmit power with different minimum QoS requirements for PS mode.

**Figure 12:**
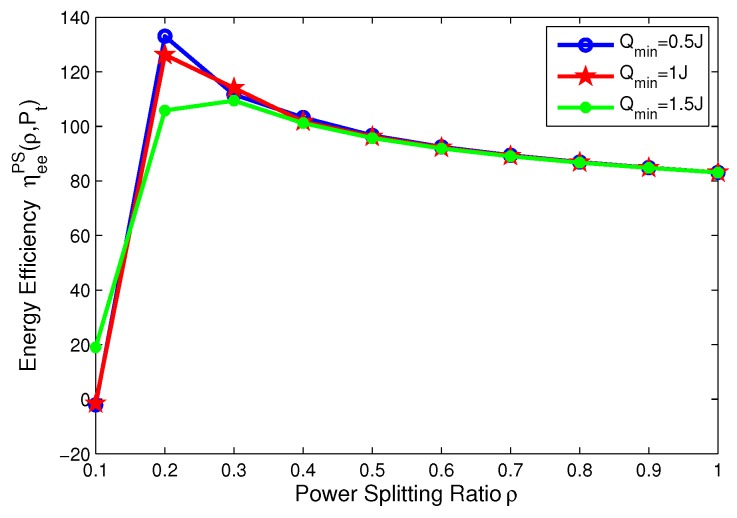
Energy efficiency versus power splitting ratio with different minimum harvested energy requirements for PS mode.

**Figure 13:**
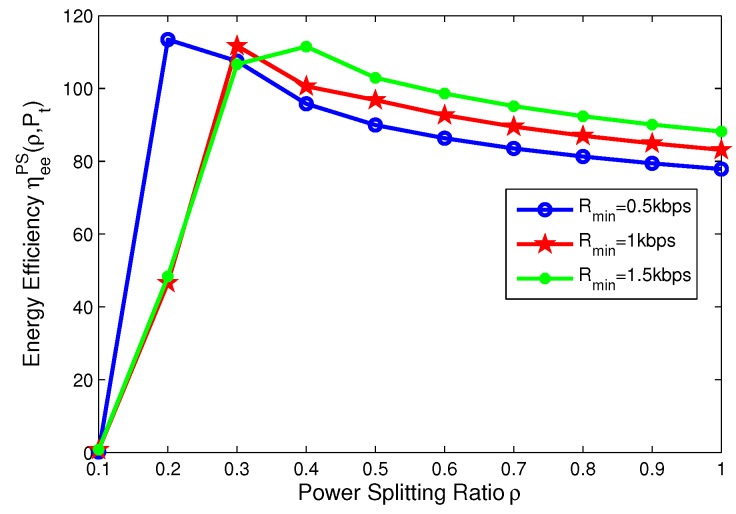
Energy efficiency versus power splitting ratio with different minimum QoS requirements for PS mode.

In this subsection, the effects of the minimum QoS and minimum harvested energy requirements on the energy efficiency are illustrated in Figure 10, Figure 11, Figure 12 and Figure 13, respectively. For PS mode, Figure 10 and Figure 11 show that the energy efficiency increases as the transmit power increases before achieving optimal peak value for different cases. Specifically, Figure 10 reveals that the larger the minimum harvested energy requirement is, the lower energy efficiency is. Moreover, Figure 13 shows that the larger minimum QoS is, the lower energy efficiency is. This is due to the fact that more power is allocated to guarantee reliable communication and harvested energy requirement. On the other hand, for PS mode, Figure 12 and Figure 13 show that the energy efficiency increases as the power splitting ratio increases when ρ is small. After energy efficiency achieves the peak value, it decreases as the power splitting ratio increases. In Figure 12, the effect of minimum harvested energy requirement on energy efficiency is shown and the larger minimum harvested energy requirement is, the lower energy efficiency is, when ρ∈[0.2,0.3]. In Figure 13, for different levels of minimum QoS, the larger the minimum QoS is, the slower energy efficiency achieves optimal peak value at a time.

### 8.4. Effects of Minimum QoS and Minimum Harvested Energy on Energy Efficiency for TS Mode

**Figure 14:**
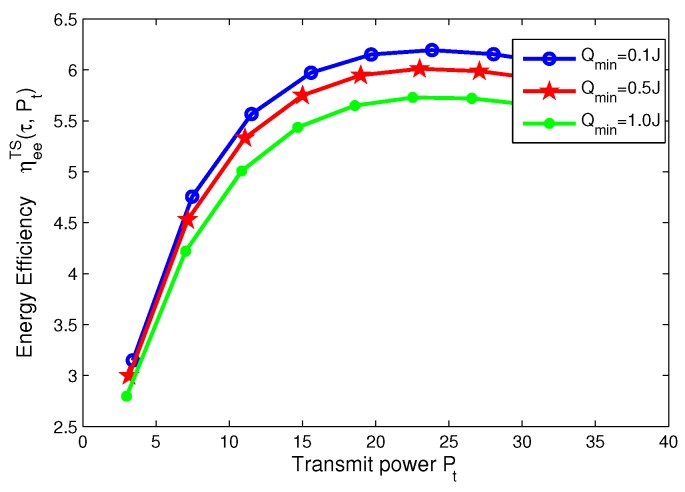
Energy efficiency versus transmit power with different minimum harvested energy requirements for TS mode.

**Figure 15:**
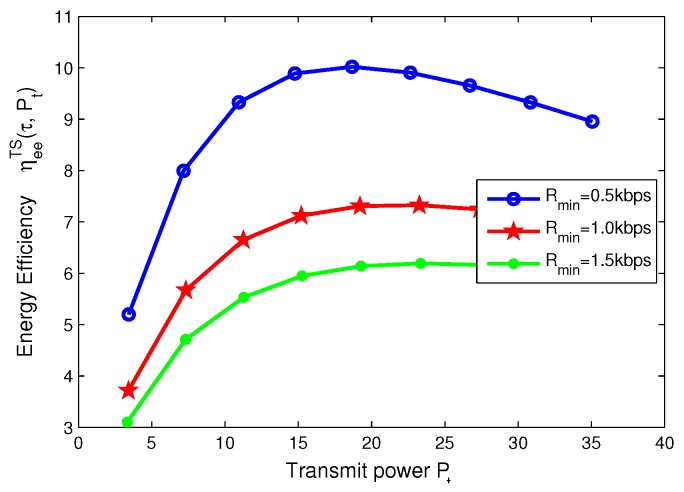
Energy efficiency versus transmit power with different minimum QoS requirements for TS mode.

**Figure 16:**
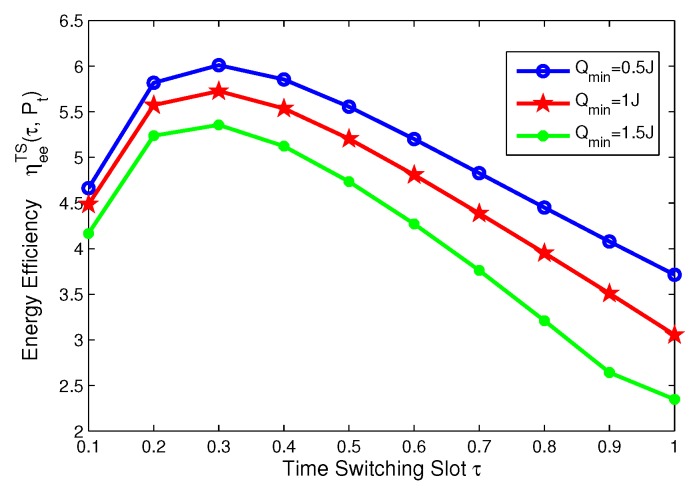
Energy efficiency versus power splitting ratio with different minimum harvested energy requirements for TS mode.

**Figure 17:**
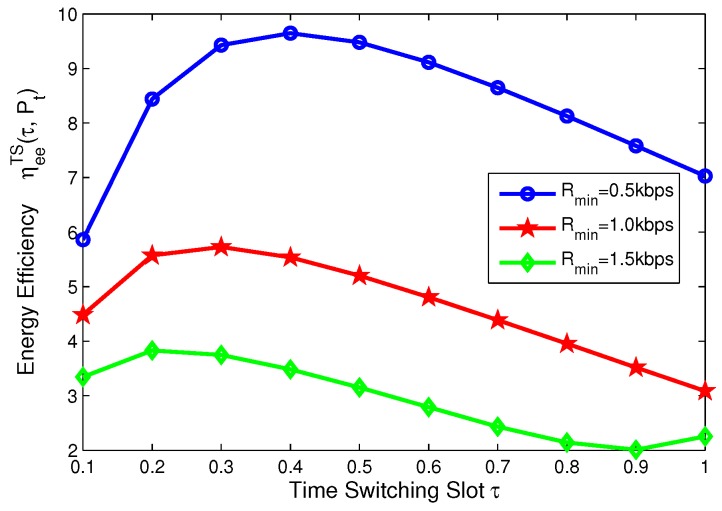
Energy efficiency versus power splitting ratio with different minimum QoS requirements for TS mode.

In this subsection, for TS mode, the effects of minimum QoS and minimum harvested energy requirement on energy efficiency are illustrated in Figure 14, Figure 15, Figure 16 and Figure 17, respectively. Specifically, in Figure 14 and Figure 15, with the increasing of transmit power, energy efficiency increases before achieving the optimal solution. It is shown from Figure 14 that the higher the minimum harvested energy requirement is, the lower energy efficiency is. In addition, Figure 15 shows that the larger minimum QoS requirement is, the lower energy efficiency is. On the other hand, in Figure 16 and Figure 17, the energy efficiency increases as the time switching slot increases before obtaining optimal value. After achieving peak value, energy efficiency decreases as time switching slot increases. In addition, Figure 16 shows that the larger the minimum harvested energy requirement is, the lower energy efficiency is as well as minimum QoS in Figure 17. These observations predict that the optimal value of time switching slot can be obtained to maximize energy efficiency of the SWIPT system by using our proposed algorithms.

### 8.5. Energy-Throughput Efficiency versus Outage Target Rate

**Figure 18:**
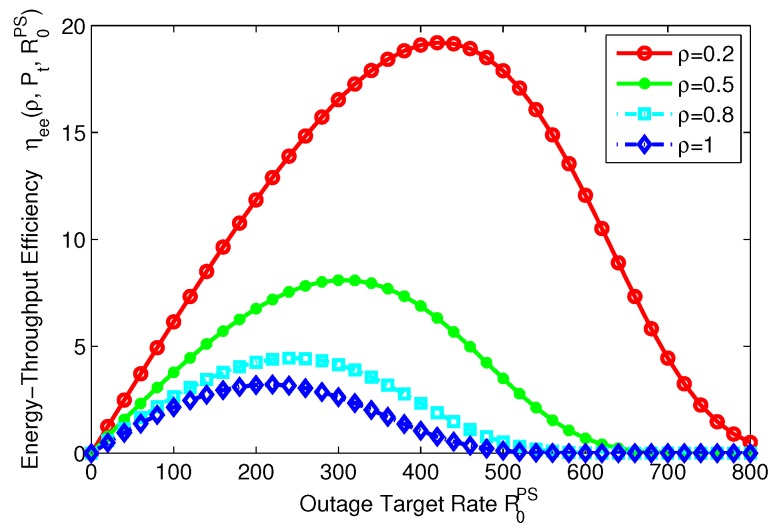
Energy-throughput efficiency versus outage target rate with different power splitting ratio for PS mode.

**Figure 19:**
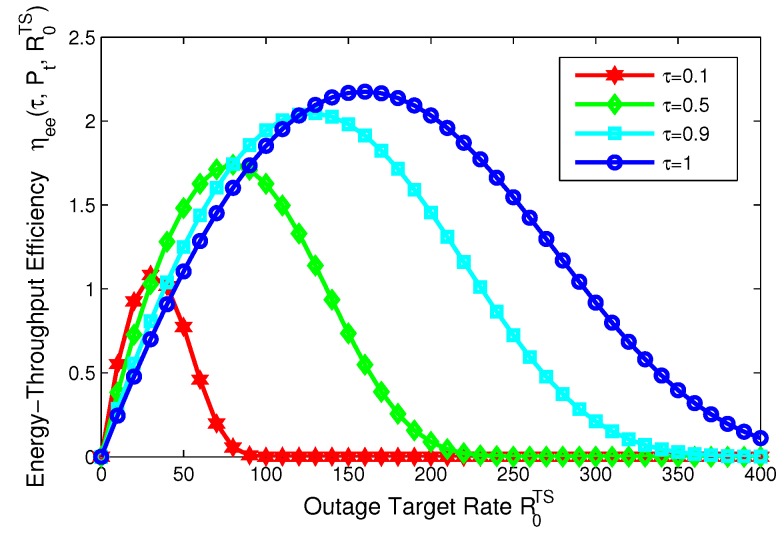
Energy-throughput efficiency versus outage target rate with different time switching slot for TS mode.

In this subsection, the relation between energy-throughput efficiency and outage target rate are illustrated for PS, TS mode in Figure 18 and Figure 19, respectively. Figure 18 shows that the energy-throughput efficiency is quasi-concave and decreases as the power splitting ratio increases. It is worth noting that the maximum value of energy-throughput efficiency is lower than the other scenarios when ρ=1, which represents the scenario without energy harvesting. This observation implies that energy-throughput efficiency can be enhanced by designing different system parameters ρ. In addition, energy-throughput efficiency maximization can be achieved for different levels of outage target rate in the SWIPT system with PS mode.

Figure 19 illustrates energy-throughput efficiency versus outage target rate with different time switching slots for TS mode. It is observed from Figure 19 that energy-throughput efficiency with respect to the outage target rate is quasi-concave. This observation means that the optimal value of outage target rate can be achieved to maximize energy-throughput efficiency for TS mode. In addition, the maximum of energy-throughput efficiency increases as the time switching slot increases. In a word, energy-throughput efficiency and outage target rate can achieve a tradeoff in the SWIPT system with PS, TS mode. From a green communication perspective, energy-throughput efficiency can be enhanced by designing a proper transceiver with different system parameters when the transmitter does not know or partially knows the CSI of the receiver.

## 9. Conclusions

In this paper, we have investigated the energy efficient transceiver design from different scenarios of green communication for WRSN with SWIPT. We have demonstrated the concavity of the energy efficiency function and quasi-concavity of the energy efficiency objective function. The energy efficient transceiver design problems for the SWIPT system with two modes are formulated as a fractional programming problem, in which the constraints of minimum QoS, minimum harvested energy requirement and maximum transmit power and circuit power consumption are taken into consideration. By exploiting the properties of nonlinear fractional programming, the proposed problems are transformed into the equivalent convex optimization with a tractable parameterized form. An efficient iterative algorithm for energy efficiency maximization is derived by Lagrangian dual decomposition. Finally, simulation results illustrate that the proposed algorithm converges to the optimal solution within a small number of iterations, which shows the achievable maximum energy efficiency in the SWIPT system for two receiver modes. Moreover, the tradeoffs between EE and SE, energy-throughput efficiency and outage target rate are observed from a green communication perspective. Our research results reflect the effects of the minimum harvesting energy requirement, minimum QoS, power splitting ratio, time switching slot and transmit power on the energy efficiency of SWIPT system. These results means that it is helpful for energy efficient transceiver design with SWIPT by optimally adjusting the system parameters in practical application. Our main contribution is to optimize the design of a smart terminal with SWIPT from an energy efficiency perspective.

From a green communication perspective, we can observe two research paradigm shifts: one is from energy saving to energy complement, and the other is from spectral efficiency to energy efficiency. Our work on the energy efficiency maximization for transceiver design of SWIPT system combines the two new research trends, i.e., energy harvesting and energy efficiency. Thus, our research work promotes a deeper understanding on SWIPT system transceiver design from a green communication perspective. Our future research can extend to the energy efficient smart terminal design with SWIPT in a massive MIMO system.

## Figures and Tables

**Table 1:** List of notations.

Notation	Definition
$P_{t}$	Transmit power
*h*	Channel power gain
ρ	Fraction of information decoding power
1−ρ	Fraction of energy harvesting power
τ	Time for information transmission
T−τ	Time for power transfer
*W*	Channel bandwidth
$P_{c}$	Static circuit power
μ	The inverse of power amplifier efficiency
ξ	Energy harvesting efficiency
$\sigma_{a}^{2}$	Antenna noise power
$\sigma_{s}^{2}$	Signal processing noise power
$P^{max}$	Maximum transmitted power
$R^{PS}$	Achievable information decoding rate for PS mode
$R^{TS}$	Achievable information decoding rate for TS mode
$Q_{EH}\left(P_{t},\rho \right)$	Energy harvesting power sum for PS mode
$Q_{total}\left(P_{t},\rho \right)$	Total power consumption for PS mode
$Q_{EH}\left(P_{t},\tau \right)$	Energy harvesting power sum for TS mode
$Q_{total}\left(P_{t},\tau \right)$	Total power consumption for TS mode
$Q_{EH}$	Energy harvesting power sum
$Q_{total}$	Total power consumption in the system
$\eta_{ee}^{PS}\left(P_{t},\rho \right)$	Energy efficiency for PS mode
$\eta_{ee}^{TS}\left(P_{t},\tau \right)$	Energy efficiency for TS mode
$R_{min}$	Minimum information decoding rate satisfied QoS
$Q_{min}$	Minimum energy harvesting requirement
$R_{0}^{PS}$	Outage target rate for PS mode
$R_{0}^{TS}$	Outage target rate for TS mode
$\eta_{ee}^{PS}\left(R_{0}^{PS},P_{t},\rho \right)$	Energy-throughput efficiency for PS mode
$\eta_{ee}^{TS}\left(R_{0}^{TS},P_{t},\tau \right)$	Energy-throughput efficiency for TS mode

**Table 2:** Simulation parameters used in this section unless otherwise specified.

Simulation Parameter	Value
Transmit power, $P_{t}$	[10,15,20,25] mW
Channel power gain, *h*	1
Fraction power for information decoding , ρ	[0.2,0.5,0.8,1]
Time for information transmission τ	[0.2,0.5,0.8,1] s
Channel bandwidth, *W*	100 MHZ
Static circuit power, $P_{c}$	10 mW
Inverse of power amplifier efficiency, μ	1,2
Energy harvesting efficiency, ξ	1
Antenna noise power, $\sigma_{a}^{2}$	0 dBm, −20 dBm
Signal processing noise power, $\sigma_{s}^{2}$	0 dBm, −20 dBm
Maximum transmitted power, $P^{max}$	30 mW
Minimum information decoding rate satisfied QoS, $R_{min}$	1 kbps
Minimum energy harvesting requirement, $Q_{min}$	0.1, 1 J
Maximum number of outer loop iterations, $L_{outer}^{D}$	20
Maximum number of inner loop iterations, $L_{inner}^{D}$	100
Convergence tolerance of iterative algorithms, $\varepsilon_{outer}^{D}=\varepsilon_{inner}^{D}$	$10^{-5}$
